# Neutrophil extracellular traps promote angiogenesis in gastric cancer

**DOI:** 10.1186/s12964-023-01196-z

**Published:** 2023-07-21

**Authors:** Shifeng Yang, Boshi Sun, Jiacheng Li, Nana Li, Ange Zhang, Xinyu Zhang, Hao Yang, Xiaoming Zou

**Affiliations:** 1 Department of General Surgery, The Second Affiliated Hospital of Harbin Medical University, No. 246 Xuefu Road, Nangang DistrictHeilongjiang Province, Harbin, 150001 China; 2 The Key Laboratory of Myocardial Ischemia, Ministry of Education, Harbin, China; 3 Department of General Surgery, The First Affiliated Hospital of Jiamusi University, Heilongjiang Province, Jiamusi, 154002 China

**Keywords:** Gastric cancer, Neutrophil extracellular traps, Angiogenesis, Proteomic techniques, CCDC25

## Abstract

Although antiangiogenic therapy has been used in gastric cancer, disease progression due to drug resistance remains common. Neutrophils play an important role in the occurrence and progression of cancer via neutrophil extracellular traps (NETs). However, few studies have investigated angiogenic regulation in gastric cancer. We aimed to determine the role of NETs in promoting angiogenesis in gastric cancer. Multiple immunohistochemical staining was used to analyze the spatial distribution of NETs and microvessels in patient tissue samples. A mouse subcutaneous tumor model was established to determine the effect of NETs on tumor growth, and changes in microvessel density were observed via immunohistochemical staining. We screened differentially expressed proteins in HUVECs stimulated by NETs via proteomics. Cell Counting Kit-8, EdU labeling, and tubule formation assays were used to verify the effect of NETs on HUVEC proliferation, migration, and tubule formation. Blocking NETs, which was related to decreased microvessel density, significantly inhibited tumor growth in the murine subcutaneous tumor model. Compared with those of the control group, tumor volume and mass among mice in the inhibition group decreased by 61.3% and 77.9%, respectively. The NET-DNA receptor CCDC25 was expressed in HUVECs, providing a platform for NETs to promote HUVEC proliferation, migration, and tubulation. In an in vitro rat aortic explant model, NETs induced HUVEC proliferation, survival, and chemotaxis, which were not significantly different from those observed in the VEGF stimulation group. Our results confirm that NETs promote angiogenesis in gastric cancer, providing a theoretical basis for identifying new anti-vascular therapeutic targets.

**Graphical Abstract:**

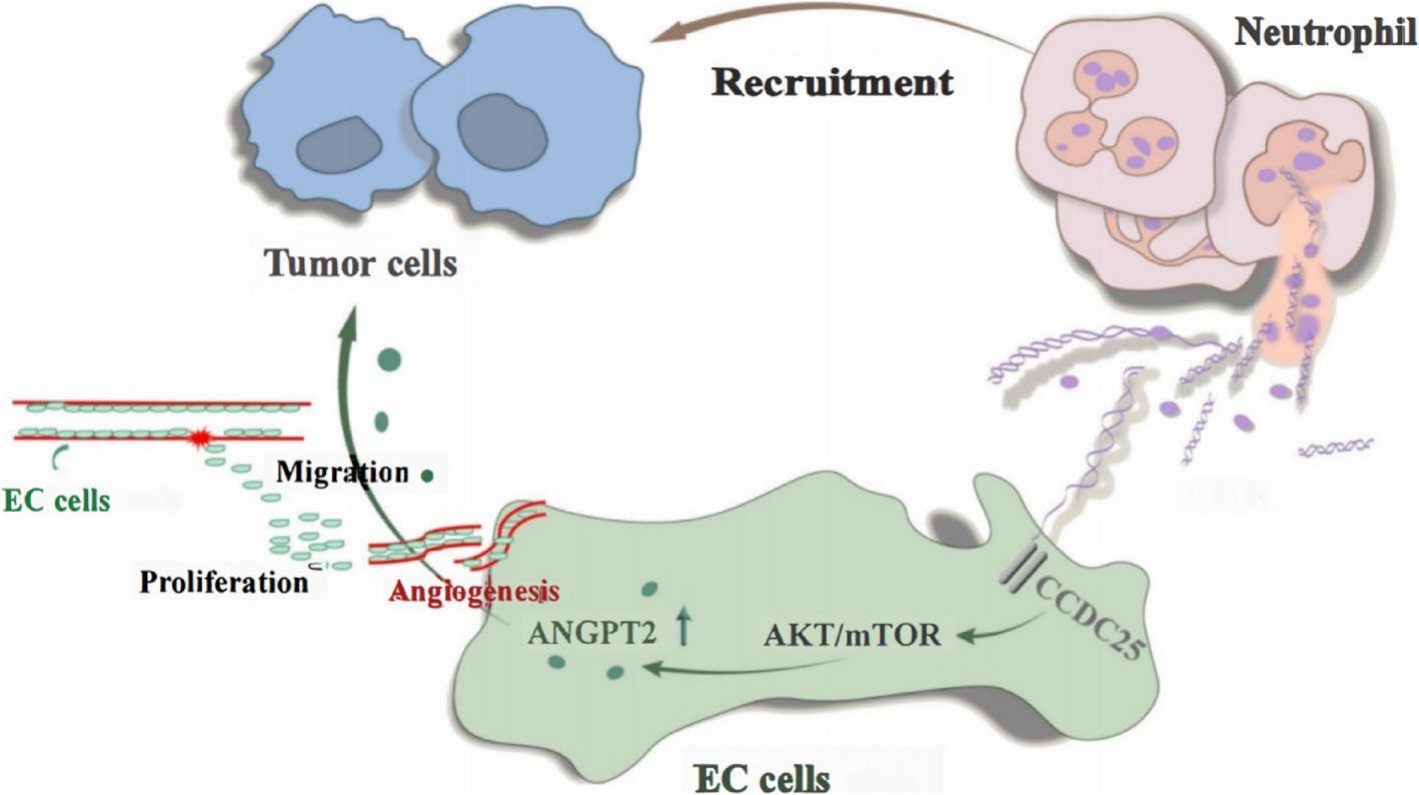

Video Abstract

**Supplementary Information:**

The online version contains supplementary material available at 10.1186/s12964-023-01196-z.

## Introduction

Angiogenesis is important for tumor progression and plays a key role in tumor growth and metastasis [1, 2]. In the 1970s, Professor Folkman proposed that tumor growth and metastasis depended on angiogenesis, and inhibition of angiogenesis could be an effective strategy for tumor treatment [3, 4]. In recent years, the identification of targeted angiogenic factors has become a popular area of research in tumor treatment and prevention [5, 6]. Presently, antiangiogenic drugs approved by the Food and Drug Administration are classified into single- and multitarget inhibitors [7]. Vascular endothelial growth factors (VEGFs) are important target molecules against tumor angiogenesis, and their inhibition has demonstrated favorable therapeutic effects in many cancers.

Gastric cancer is a common malignancy of the digestive system characterized by strong heterogeneity, treatment refractoriness, and a poor prognosis [8, 9]. Anti-VEGF therapy has been used in gastric cancer and has shown favorable clinical results [10–12]; however, similar to the results of other treatments, some patients eventually develop resistance to VEGF therapy [13]. Therefore, other targets must be explored to inhibit angiogenesis and provide alternative effective treatments.

Studies have suggested that tumor angiogenesis results from the interaction of various cells and factors, including tumor cells and endothelial cells [14, 15]. Immune cells, such as macrophages, are recruited and promote angiogenesis in the tumor microenvironment in some patients who are resistant to VEGF inhibitors [16]. In a transplanted tumor model of Lewis lung cancer, Tie2 activation was found to induce normalization of the tumor vasculature, enhance blood perfusion and chemotherapeutic drug release, significantly reduce lactic acidosis, and reduce tumor growth and metastasis. Simultaneously activating Tie2 and inhibiting Ang2 is an effective treatment strategy for enhancing chemotherapeutic drug entry into tumors [17].

Although several immune cell types infiltrate the tumor microenvironment, their distribution and function differ depending on the tumor type [18, 19]. Previously, neutrophils were considered the first line of defense against pathogens and inflammatory cells and were necessary for maintaining normal immune function [20]. However, recent studies have contradicted the traditional perception regarding neutrophils by demonstrating their immunosuppressive function in tumors. Many studies have shown that neutrophils can fight malignancies, although detailed mechanistic insights are lacking. For example, in colon cancer, high neutrophil counts were related to a good prognosis [21], and in breast cancer models, depletion of neutrophils promoted disease progression [22]. Macrophages can activate neutrophils through interferons to secrete chemokines and cytokines that activate the immune system [23]. Neutrophils can also directly attack cancer cells by producing ROS and ATP [24]. Gentles et al. analyzed the density of immune cell groups and survival times of patients with 39 cancer types, including 3,000 solid tumors, and found that the group with high-density neutrophil infiltration had the most unfavorable prognosis [25]. Increased neutrophil levels play an important role in the prognosis of cancer patients because neutrophils can inhibit the antitumor immune response.

Neutrophil extracellular traps (NETs) are special structures based on neutrophils [26]. Recent studies have shown that NETs play an important role in promoting tumor progression [27, 28]. In our previous study, extensive neutrophil infiltration and NET deposition in gastric cancer were observed, both of which were associated with poor prognoses. Moreover, in vitro, we found that NETs promoted invasion, metastasis, and epithelial-mesenchymal transformation (EMT) in gastric cancer cells and stimulated endothelial cells to release tissue factors that activate the coagulation pathway without significantly affecting cell proliferation [29–31]. Thus, antagonizing NETs could significantly inhibit tumor growth related to a decrease in microvessel density in subcutaneous tumor models of animals. Therefore, we aimed to investigate the effects of NETs on endothelial cell function and angiogenesis in gastric cancer patients using proteomics to explore how neutrophils promote cancer progression and to provide a basis for the development of new therapies against tumor angiogenesis.

## Materials and methods

### Patients and tissue samples

This study included 20 healthy participants and 60 primary gastric cancer patients newly diagnosed via pathological examination who were admitted to the Second Affiliated Hospital of Harbin Medical University between October 2019 and April 2021. We based the tumor, node, and metastasis evaluation, staging, and histological classification of gastric cancer on the 8th edition of the American Joint Commission on Cancer staging manual [32]. The inclusion criteria were patients aged 18–65 years without any endocrine, cardiovascular, hematologic, or infectious diseases. The exclusion criteria were pregnancy, coexisting cancers, and the receipt of preoperative antineoplastic treatment. Samples of cancerous and paracarcinoma tissues of gastric cancer patients were collected. All patients provided informed consent, and the study protocol was approved by the Internal Audit and Ethics Committee of the Second Affiliated Hospital of Harbin Medical University (approval number: KY2021-075).

### Isolation of neutrophils

A neutrophil isolation kit (TBD Sciences, Tianjin, China) was used to isolate neutrophils from blood samples. Venous blood samples were collected using a 5 mL catheter containing 3.2% sodium citrate. A 5 mL neutrophil separation solution (anticoagulant) was then mixed with whole blood. The samples were centrifuged at 450 × g for 40 min at 20 °C. The neutrophil layer was added to the erythrocyte separation solution and repeatedly centrifuged at 450 × g at 24 °C for 5 min until the red blood cells disappeared. The neutrophils were collected and placed in a container with 1 mL of phosphate-buffered saline (PBS).

### Formation, separation and preparation of NETs

Purified neutrophils were inoculated in a six-well plate (1 × 10^6^ cells/well), stimulated with 100 nM phorbol myristate acetate (HY-18739; MedChemExpress LLC, USA), and cultured in a 5% CO_2_ incubator for 4 h at 37 °C. The upper medium was then gently suctioned, leaving NETs and neutrophils. Precooled PBS without calcium and magnesium was added, and the NETs and neutrophils attached to the bottom layer were eluted. The liquid from the six-well plate was collected and centrifuged at 450 × g for 10 min at 4 °C, and the supernatant was collected and centrifuged at 15,000 × g for 15 min at 4 °C. The supernatant was discarded, and the precipitate was resuspended in PBS. The concentration was determined using a micro-DNA instrument (206–26,300-48; BioSpec-nano, Japan), and the sample was stored at -20 °C.

### Cell culture

The human metastatic gastric cancer cell lines MKN-45 and MKN-1, human primary gastric cancer cell lines AGS and HGC27, and human gastric epithelial cells (GES-1) were all purchased from Procell (Wuhan, China). Primary human umbilical vein endothelial cells (HUVECs) and primary human pulmonary microvascular endothelial cells (HMVECs) were purchased from San Diego (HTX1922, USA). All cell lines were verified via short tandem repeat mapping and negative detection of mycoplasma. All cell lines were cultured in RPMI 1640 (Gibco, USA) and endothelial cell medium (ScienCell Research Laboratories, USA) containing 10% fetal bovine serum at 37 °C in a 5% CO_2_ incubator. The medium without fetal bovine serum was changed according to the experimental design and cultured for 48 h for further analysis.

### Treatment of endothelial cells

In the NET stimulation group, HUVECs were cultured in medium containing different concentrations of NETs (from gastric cancer patients) at 37 °C in a 5% CO_2_ incubator for 24 h. In the DNase I inhibition group, isolated NETs were pretreated with DNase I (100 U/mL) (Thermo Fisher Scientific, USA) at 37 °C for 1 h and cocultured with HUVECs for 24 h. The same amount of PBS was added in the negative control group.

### Tandem mass tag tandem mass spectrometry labeling

Trypsin-hydrolyzed peptides were desalted with Strata X C18 (Phenomenex, USA) and freeze-dried in a vacuum. Peptides were dissolved with 0.5 M triethylammonium bicarbonate and labeled according to the operating instructions of the tandem mass tag (TMT) kit. Briefly, after the labeled reagent was thawed, it was dissolved in acetonitrile, mixed with the peptide, and incubated at room temperature for 2 h. The labeled peptide was then mixed, desalted, and vacuum freeze-dried.

### Immunohistochemistry

Gastric paracarcinoma and cancerous tissues from animal models were fixed with 4% paraformaldehyde for 48 h, dehydrated, and embedded in paraffin. The paraffin sections were subjected to antigen retrieval, sealing, incubation with primary antibodies (1:50 dilutions of myeloperoxidase [MPO] [ab90810; Abcam, UK], cit-H3 [ab5103; Abcam], CD66b [ab207718; Abcam], CCDC25 [21209–1-AP; Proteintech, China], and CD31 [ab228968; Abcam]), incubation with secondary antibodies (1:100 dilutions of horseradish peroxidase [HRP]-labeled goat anti-mouse IgG and HRP-labeled goat anti-rabbit IgG), 4′,6-diamidino-2-phenylindole (DAPI) staining (Solarbio Life Science, Beijing, China), coloration, and sealing. The samples were then observed and photographed using a confocal microscope (LSM800; Zeiss, Germany).

### Hematoxylin–eosin

For dewaxing, slides were placed in xylene I for 10 min, xylene II for 10 min, anhydrous ethanol I for 15 min, anhydrous ethanol II for 5 min, 95% alcohol for 5 min, 90% alcohol for 5 min, 80% alcohol for 5 min, 70% alcohol for 5 min and distilled water washes. For hematoxylin staining of the nucleus, slides were stained with hematoxylin for 5 min, followed by tap water rinses, incubation in differentiation solution for several seconds, tap water rinses until the samples turned blue again, and a running water rinse. For eosin staining of the cytoplasm, eosin staining solution was added for 3 min, slides were dehydrated and sealed, and the slides were then placed in 95% alcohol I for a minute, 95% alcohol II for 5 min, anhydrous ethanol I for 5 min, anhydrous ethanol II for 5 min, xylene I for 5 min, and xylene II for dehydration and transparency in 5 min. The glass slides were taken from the xylene to dry slightly, sealed in neutral gum, observed and used to take photos.

### Immunofluorescence assay

HUVECs were stimulated with 0.4 μg/mL NETs for 24 h. The sample was removed from the 24-well plate, fixed with 1% paraformaldehyde for 15 min, washed twice with PBS, incubated with 3% bovine serum albumin for 30 min, and then washed twice with PBS. The primary antibodies, 1:1000 dilutions of anti-ZO-1 (ab190085; Abcam), anti-VE (ab33168; Abcam), anti-CD31 (ab228968; Abcam), anti-ANGPT2 (Df6137; Affinity, China), anti-von Willebrand factor (vWF) (ab154193; Abcam), or anti-CCDC25 (21,209–1-AP; Proteintech), were incubated overnight at 4 °C. The secondary antibodies, labeled with Alexa Fluor 488 and 594 (1:200; Abcam, USA), were incubated for 30 min and washed twice with PBS. Cytoskeletal staining was conducted as follows: the ghost pen cyclopeptide was incubated for 10 min and washed twice with PBS. Nuclear staining was performed via incubation with DAPI (Solarbio Life Science) for 10 min, followed by washing twice with PBS. Sharp tweezers were used to remove the slide from a 24-well plate and affix the cellular side upside down onto the glass slide. Glycerol was added for anti-quenching, and the slides were observed and photographed using a confocal microscope.

### Western blotting

When the growth density of HUVECs reached 80%, the cells were stimulated with 0, 0.2, 0.4, and 0.8 μg/mL NETs for 24 h. The cell protein was then extracted, and the expression of ZO-1 (ab190085; Abcam), VE (ab33168; Abcam), AKT (ab38449; Abcam), p-AKT (ab8805; Abcam), mTOR, and p-mTOR (ab32028; Abcam) was analyzed via western blotting.

### Tubule formation experiment

Matrigel (BD356234; Corning, USA) was placed on ice, oscillated, and mixed using a vortex instrument to avoid delamination. Then, 100 μL of Matrigel was added to each well of a precooled 24-well plate. Matrigel was incubated at 37 °C for 45 min until it solidified. The pretreated HUVECs were digested with trypsin, centrifuged, resuspended, and counted. The concentration of the cell suspension was adjusted to 4 × 10^5^ cells/mL. A 100 μL cell suspension was added to each of the three wells of each group: control, NETs + and NETs + DNase I. After incubation at 37 °C, tubules formed after 4 h were photographed using a microscope.

### Arterial ring angiogenesis experiment in SD female rats

Female Sprague Dawley rats weighing 150 g were anesthetized, euthanized with cervical dislocation, and immersed in 75% alcohol for 10 min. The thorax and abdomen were incised, the aorta was peeled and placed in complete medium, and the adventitia was removed with microscopic forceps and cut into 2 mm vascular segments. Then, 100 μL of Matrigel was added to a 24-well plate, and the vascular segments were embedded in Matrigel. Matrigel was incubated at 37 °C for 45 min until it solidified. The conditioned medium was then added, cultured in an incubator at 37 °C, and changed after 2–3 days. Photographs were then obtained with a microscope after 7 days. Neovascularization was determined and analyzed using ImageJ software (National Institutes of Health, MD, USA).

### Cell Counting Kit-8 assay

A 100 μL cell suspension pretreated with PBS and 0, 0.2, 0.4, or 0.8 μg/mL NETs was inoculated in a 96-well plate (six plates/group) at a cell density of 5,000 per well. The plates were cultured at 37 °C in a 5% CO_2_ incubator for 24, 48, 72, or 96 h. Then, 10 μL of Cell Counting Kit-8 (CCK-8) solution (abs50003; Absin Bioscience, Inc., China) was added to each plate at a fixed time point, and the culture plate was incubated for 2 h. The absorbance at 450 nm was measured using an enzyme labeling instrument, and data were collected and statistically analyzed using GraphPad Prism version 8.0 (GraphPad Software, Inc., CA, USA).

### EdU (5-ethynyl-2′-deoxyuridine) assay

In a 96-well plate, 2 × 10^4^ cells in the logarithmic growth phase were inoculated and cultured to the normal growth stage. After coculture with HUVECs in medium containing PBS and 0.4 μg/mL NETs or 0.4 μg/mL NETs + DNase I for 24 h, the cells were labeled with 5-ethynyl-2′-deoxyuridine (EdU) (Cell-Light EdU Apollo 567 kit; RiboBio, China), fixed, observed, and photographed using a fluorescence microscope.

### Characterization of NETs by scanning electron microscopy

Neutrophils cultured on the cover slides were stimulated with 0.4 μg/mL NETs for 24 h, washed, and fixed with 2.5% glutaraldehyde. The samples were then washed with PBS, treated with osmium tetroxide and dehydrated with 30%, 50%, 90%, 95%, and 100% ethanol concentrations at 10 min/gradient. After 20 min of pure acetone replacement, the neutrophils were transferred into isoamyl acetate in the intermediate solution. After critical point drying, the surface of the sample was coated with a thick platinum layer, which was observed and photographed using a Smur3400N electron microscope (Hitachi, Japan).

### Animal model construction

The animal experiment was carried out at the Animal Experimental Center of the Key Laboratory of Myocardial Ischemia of the Second Affiliated Hospital of Harbin Medical University in strict accordance with the scheme approved by the Animal Care and Use Committee (approval no. SYDW2021-072). Ten BALB/c nude mice aged 5–7 weeks were purchased from Weitong Lihua Experimental Animal Technology Co., Ltd. (China), and kept in a 22 °C aseptic animal house. Food and autoclaved water were provided. The mice were randomly divided into the control (*n* = 5) and DNase I treatment (*n* = 5) groups. All mice were anesthetized with a 2% isoflurane mixture, and their axillary skin was disinfected with sterilized cotton balls. Then, 1 × 10^6^ HGC-27 gastric cancer cells were subcutaneously injected into the armpit, and cotton swabs were applied to stop the bleeding. The mice in the treatment group were intraperitoneally injected with deoxyribonuclease (DNase I, 50 μg/mouse, Roche) every 12 h until they were euthanized. The tumor volume in each mouse was determined once every 3 days using the following formula: 0.5 × length × width^2^. All mice were euthanized after 18 days. Tumor tissues were removed, weighed, soaked in 4% paraformaldehyde for 24 h, embedded in paraffin, and divided into 4 μm paraffin sections for follow-up immunohistochemical staining.

### Immune infiltration

The CIBERSORT algorithm provided by the IOBR R package was used to calculate the scores of immune cells in 22 types of tumor microenvironments using the default parameters. Based on the gene expression profile in the TCGA-STAD data, the proportion of immune cell infiltration was calculated.

### Drug sensitivity

With the pRRophetic R package and the expression data of model genes, the sensitivity (IC50 value) of 138 drugs in the GDSC database was predicted, and the sensitivity of STAD patients to drug therapy was evaluated based on the predicted IC50 values.

### TIDE scoring

The difference in ANGPT2 between GC samples and noncancer samples was analyzed in TCGA and GTEx. We estimated the ANGPT2 of 388 patients in the TCGA-STAD dataset and then sorted patients into high ANGPT2 and low ANGPT2 groups according to the P value of the best cut off. The online tool for TIDE scoring (http://tide.dfci.harvard.edu/login/) was used [33].

### Data analysis

The data are presented as the means ± standard deviations. We used the Shapiro–Wilk test to assess the normality of distribution of continuous variables. Multiple groups were compared using one-way analysis of variance, and paired variables were compared via paired *t* tests. All data were processed using GraphPad Prism version 8.0 (GraphPad Software, Inc.) and SPSS version 16.0 (IBM Corporation, USA). Significance was defined at p < 0.05.

## Results

### NET deposition in tissue and plasma and increased microvessel formation in gastric cancer

**Fig. 1:**
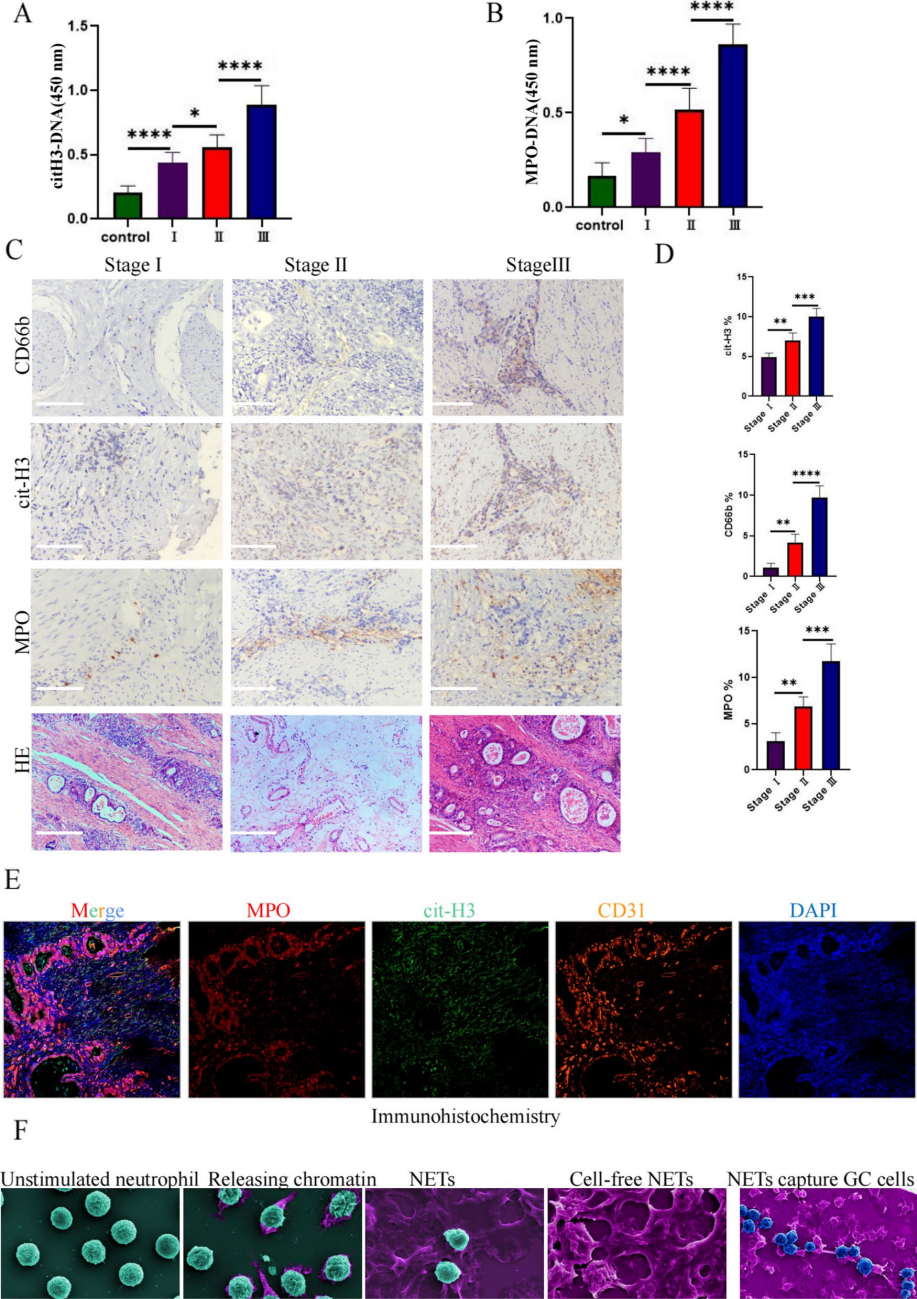
NETs in the tissues and plasma of gastric cancer patients. **a, b** Plasma levels of the NET markers citrullinated histone H3-DNA (cit-H3-DNA) and myeloperoxidase DNA (MPO-DNA) in gastric cancer (GC) patients and healthy individuals measured via ELISA. Healthy individuals, *n* = 20; stage I, *n* = 20; II, *n* = 20; III, *n* = 20. **c** The levels of neutrophils and neutrophil extracellular trap (NET) markers CD66b, cit-H3, and MPO in gastric cancer tissues were measured via immunohistochemical staining, and the vascular abundance was observed via hematoxylin and eosin (HE) staining. **d** The expression area coverage of CD66b, cit-H3, and MPO was assessed via immunohistochemical staining. **e** Analysis of the spatial distribution of the NET markers MPO and cit-H3 and the vascular marker protein CD31 via multiple immunohistochemical staining. **f** The release of NETs by neutrophils and the adhesion of NETs to gastric cancer cells were observed using a scanning electron microscope. Image J software was used for the statistical analysis. All values are presented as means ± standard deviations. *p < 0.05; **p < 0.01; ***p < 0.001; ****p < 0.0001

Analysis of NET-DNA levels in the plasma of healthy volunteers and gastric cancer patients via enzyme-linked immunosorbent assays revealed higher levels of cit-H3-DNA and MPO-DNA in the plasma of the gastric cancer patients than in the healthy individuals and higher NET-DNA deposition in patients with stage III gastric cancer than in patients with stage I and II gastric cancer (Fig. 1a, b). To study NET deposition in gastric cancer tissues, we performed immunohistochemical analyses of clinical specimens from the patients with stage I (*n* = 20), II (*n* = 20), and III (*n* = 20) gastric cancer and the corresponding adjacent tissues. Neutrophil infiltration and NET deposition occurred, and the samples were positive for MPO, cit-H3, and CD66b (Fig. 1c). Moreover, hematoxylin–eosin staining showed many microvessels in the tumor tissue (Fig. 1c). The expression area coverage of CD66b, cit-H3, and MPO identified by immunohistochemical staining in different stages showed significant differences (Fig. 1d). Multiplex immunohistochemistry showed highly consistent spatial localization of the NET markers cit-H3, MPO, and CD31 (Fig. 1e). Moreover, morphological changes in neutrophils during different stages of NET release were observed via electron microscopy (Fig. 1f).

### Neutrophils from gastric cancer patients release NETs more easily than those from healthy individuals

**Fig. 2:**
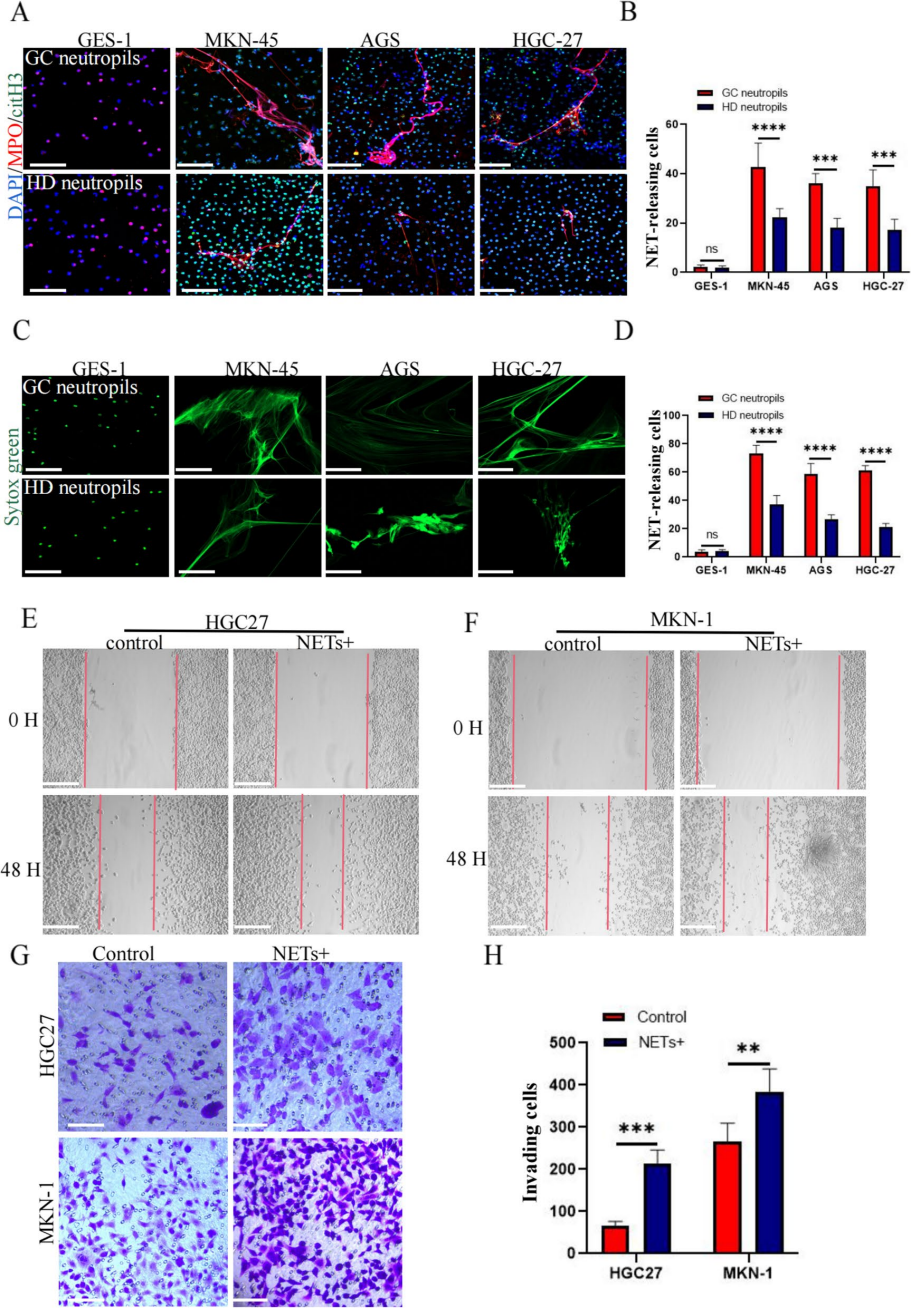
GC cells activate neutrophils to release NETs, which promote the invasion and metastasis of GC cells. **a** Immunofluorescence staining was used to observe the difference in NET release from neutrophils between healthy volunteers and gastric cancer patients under the same conditions of stimulation. Neutrophils were stimulated with the supernatants of GES-1, MKN-45, AGS, and HGC-27 cells and characterized by cit-H3 (green) and MPO (red). **b** The percentage of NET cells released by NETs. **c** Cell-impermeable SYTOX Green staining was used to observe the difference in NETs released from healthy volunteers and gastric cancer patients under the same conditions of stimulation. Neutrophils and NETs (green). **d** The percentage of NET cells released by neutrophils. **e**, **f** The effect of NETs on the migration ability of the gastric cancer cell lines MKN-1 and HGC27 was detected using the scratch test. **g** The Transwell assay was used to detect the effect of NETs on the invasive ability of the gastric cancer cell lines MKN-1 and HGC27. **h** The number of cells passing through the compartment was counted using Image J software. Magnification, × 20; scale bars: 50 μm; red, cit-H3; green, MPO; blue, DAPI. All values are presented as means ± standard deviations. **p < 0.01; ***p < 0.001; ****p < 0.0001. GC, gastric cancer; GES-1, gastric mucosal epithelial cells; NET, neutrophil extracellular trap; MPO, myeloperoxidase; cit-H3, citrullinated histone H3

To compare the ability of neutrophils to release NETs between healthy volunteers and gastric cancer patients, we isolated and extracted neutrophils from whole blood samples of patients with stage III gastric cancer and healthy volunteers. GES-1, the metastatic gastric cancer cell line MKN-45, the gastric cancer in situ cell line AGS, and HGC27-conditioned media were used as stimulants to assess NET release via immunofluorescence. The analysis showed that neutrophils from gastric cancer patients released NETs more easily under the same conditions and that the effect of stimulation of the tumor cell supernatant was stronger than that of GES-1 (Fig. 2a–d).

### NETs promote invasion and metastasis of gastric cancer cells

NETs extracted from gastric cancer patients were treated with the gastric cancer cell lines HGC27 and MKN-1. The results of the scratch test showed that NETs could significantly promote migration. The transwell assay showed that NETs could significantly promote the invasion of gastric cancer cells (Fig. 2e–h). However, CCK-8 and plate cloning experiments showed that NETs had no obvious effects on the proliferation of gastric cancer cells (Fig. S1a,b).

### *NET inhibition can suppress tumor growth *via* decreased microvessel density in animal models*

**Fig. 3:**
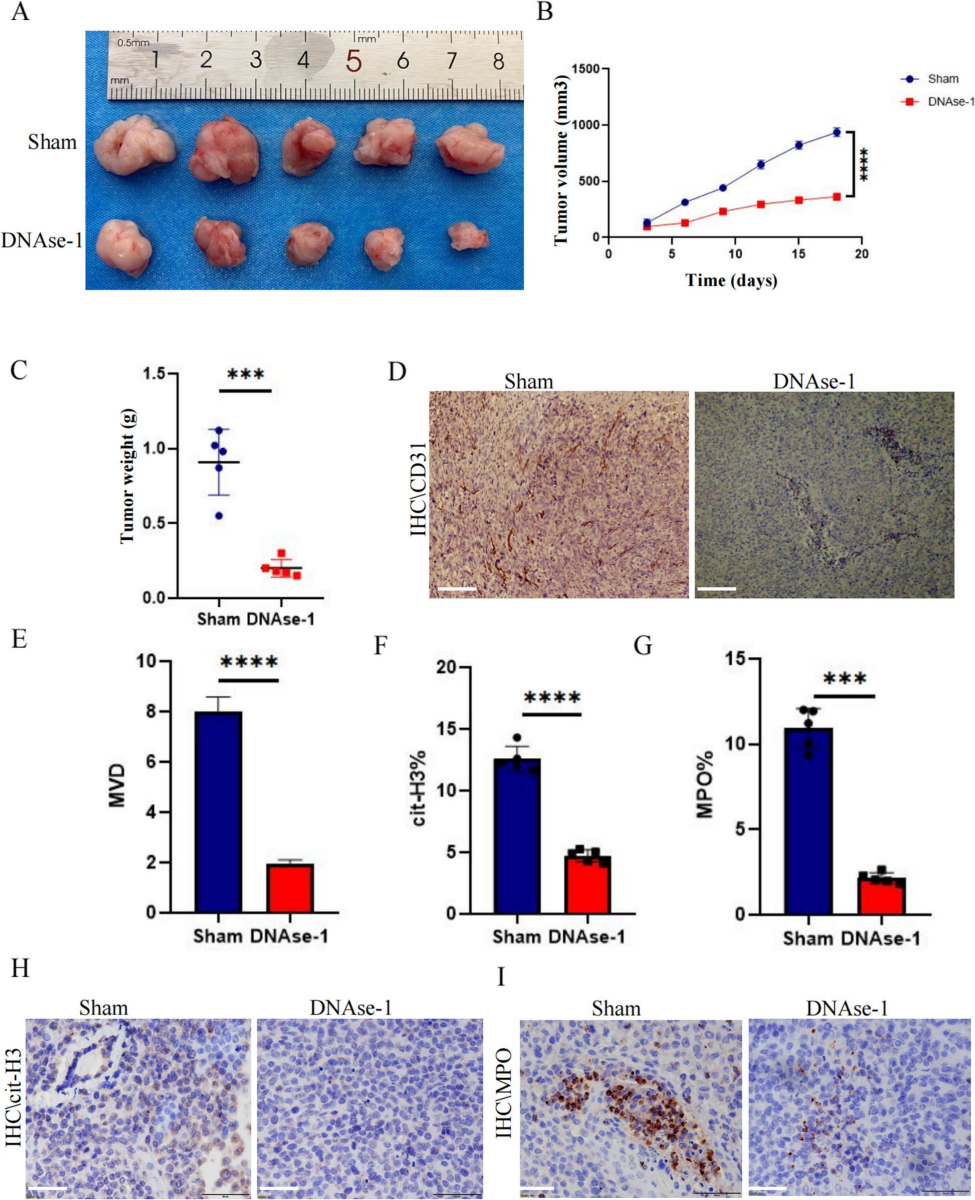
DNase-1 inhibited tumor growth in relation to a decrease in microvessel density. **a** Subcutaneous tumor samples from nude mice treated with DNase-1 and the control group (*n* = 5). **b** Growth curve of subcutaneous tumor in nude mice; all values are presented as means ± SDs. ****p < 0.0001. **c** Subcutaneous tumor sample weights; all values are presented as means ± SDs. ***p < 0.001. **d** Microvessel densities in tumor tissues of the control and DNase-1 treatment groups as assessed by immunohistochemical staining. CD31 is a positive index; magnification × 20; scale bars: 50 μm. **e** The microvessel density was calculated using Image J software; all values are presented as means ± SDs, ****p < 0.0001. **h, i** The levels of NETs in tumor tissues of the control and DNase-1 treatment groups were measured via immunohistochemical staining. cit-H3 and MPO are positive markers; magnification × 20; scale bars: 50 μm. **f, g** Statistics of the NET coverage area were calculated using Image J software. NET, neutrophil extracellular trap; MPO, myeloperoxidase; cit-H3, citrullinated histone H3; SD, standard deviation

As the cell experiments showed that NETs had no obvious effect on the proliferation of gastric cancer cell lines, we examined whether NETs played a role in tumor angiogenesis using a murine tumor model. Intraperitoneal injection of DNase I reduced the growth volume and weight of subcutaneous gastric cancer tumors compared with those of the control group (Fig. 3a, c). The growth curve showed that DNase I treatment significantly inhibited the growth rate of tumors (Fig. 3b). Having found that NETs had no significant effect on the proliferation of gastric cancer cells in vitro, we explored whether NETs could promote tumor growth by increasing angiogenesis. We found a lower positivity rate of CD31 in the tumor tissues injected with DNase I, with less NET infiltration into the tumor tissue. Positive staining of cit-H3 and MPO was used as a marker (Fig. 3d, h, i) and showed significant differences (Fig. 3e–g).

### Proteomic analysis of differential protein expression induced by NET stimulation in endothelial cells

**Fig. 4:**
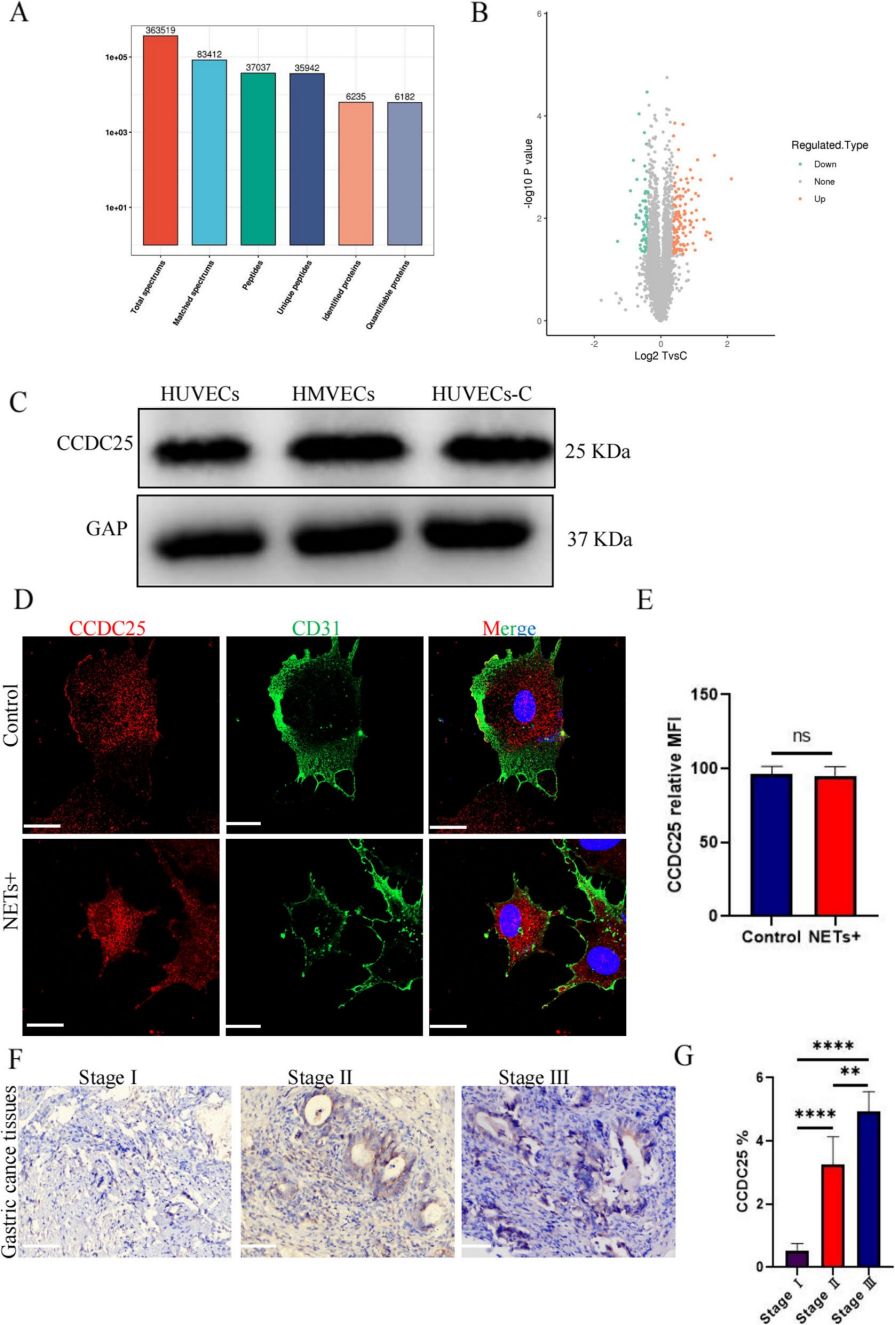
HUVECs express CCDC25, as evaluated using proteomics **a** The quantifiable proteins differentially expressed after NET stimulation of HUVECs were identified from TMT-MS/MS spectra. **b** Volcanogram of changes in relative protein abundance between NET-stimulated and unstimulated groups. **c** The expression of CCDC25 in different endothelial cells, HUVECs, HUVECs-C, and HMVECs, as detected by western blotting. **d** Differences in the expression and location of CCDC25 before and after NET stimulation as observed via immunofluorescence. **e** The average fluorescence intensity of CCDC25 was calculated using Image J software.** f** The expression of CCDC25 in stage I, II, and III gastric cancer tissues was observed by immunohistochemical staining.** g** The area covered by CCDC25 expression in immunohistochemical staining. Magnification, × 20; scale bars: 50 μm; red, CCDC25; green, CD31; blue, DAPI. All values are presented as means ± standard deviations. **p < 0.01; ****p < 0.0001 CCDC25, coiled-coil domain containing 25; HUVEC, human umbilical vein endothelial cell; HMVEC, human pulmonary microvascular endothelial cell; NET, neutrophil extracellular trap; HUVEC-C, human pulmonary microvascular endothelial cell line

To explore the specific effect of NETs on HUVECs, we subjected unstimulated HUVECs and HUVECs stimulated with NETs to tandem mass tag-tandem mass spectrometry (TMT-MS/MS) analysis and collected a total of 363,519 secondary mass spectra. Among them, there were 83,412 effective mass spectra, with a utilization rate of 22.9% (Fig. 4a). Among the quantifiable proteins, 123 upregulated proteins and 73 downregulated proteins were observed in the NET-stimulated group but not in the unstimulated group (Fig. 4b).

### The NET-DNA receptor CCDC25 is expressed in HUVECs

Using proteomics, we found that HUVECs expressed CCDC25 (Table S1). Based on the histological results, we performed basic experiments and obtained the following results. Western blotting identified CCDC25 expression in HUVECs, HUVEC-C, and HMVECs (Fig. 4c). Immunofluorescence analysis of CCDC25 localization in endothelial cells showed that CCDC25 was a transmembrane protein whose transfer into the cytoplasm increased after NET stimulation (Fig. 4d, e). Furthermore, immunohistochemical staining showed that cancer tissues expressed more CCDC25 receptors than paracarcinoma tissues (Fig. 4f, g).

**Fig. 5:**
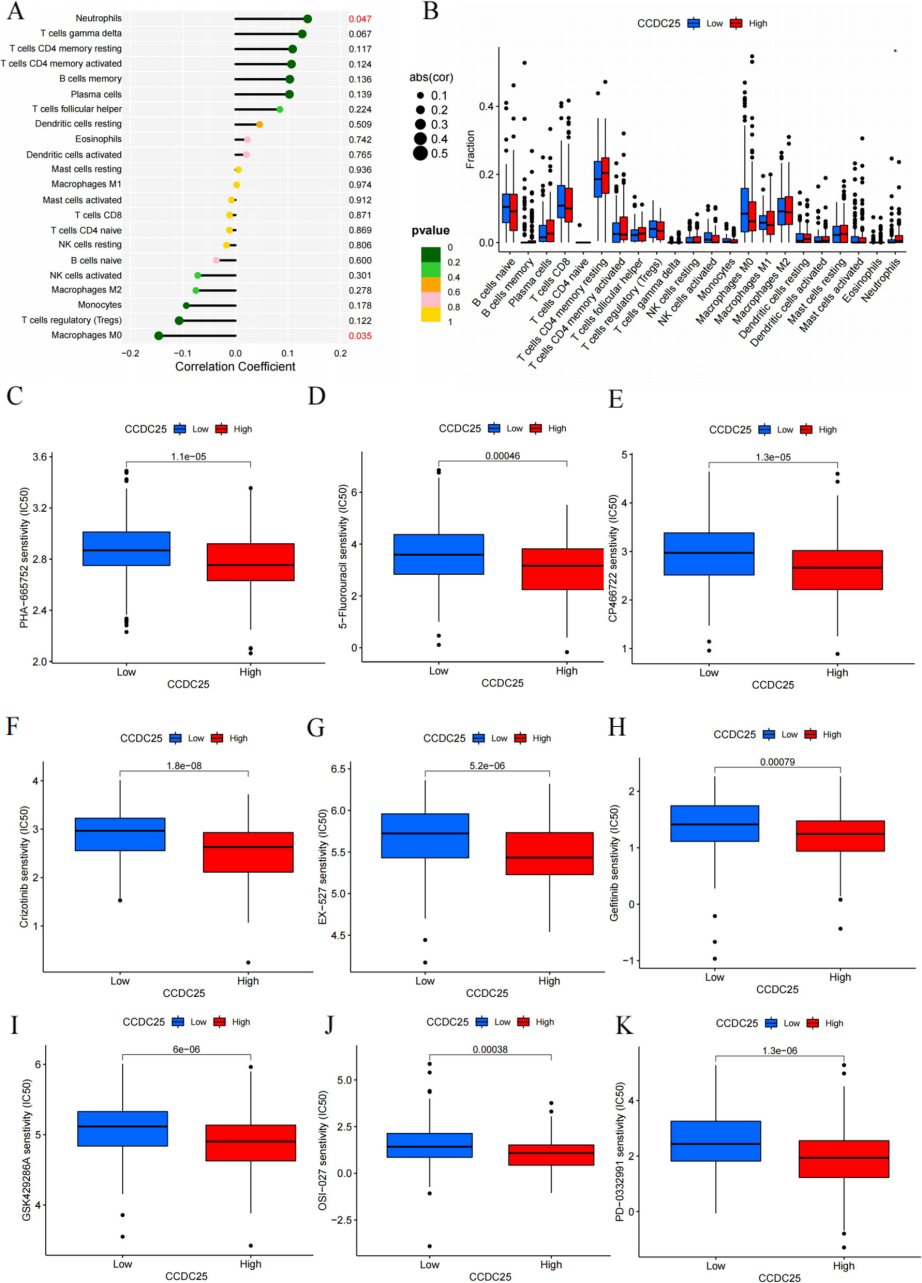
Bioinformatics analysis of the biological function of CCDC25 in gastric cancer. **a**,** b** The relationship between CCDC25 and immune cell infiltration. **c**–**k** The relationship between high and low expression of CCDC25 and sensitivity to chemotherapeutic drugs and immunosuppressants in gastric cancer patients

We further analyzed the biological function of CCDC25 in gastric cancer using bioinformatics. In the immune infiltration analysis, CCDC25 only showed statistical significance with neutrophil infiltration (Fig. 5a, b), which was highly consistent with our experimental results. We further analyzed the differences in chemosensitivity and targeted drug sensitivity in gastric cancer patients with high and low CCDC25 expression. The results showed that patients with low CCDC25 expression experienced better effects from a variety of chemotherapeutic drugs and immunosuppressants (Fig. 5c–k).

### NETs promote endothelial cell proliferation, migration and tubule formation

**Fig. 6:**
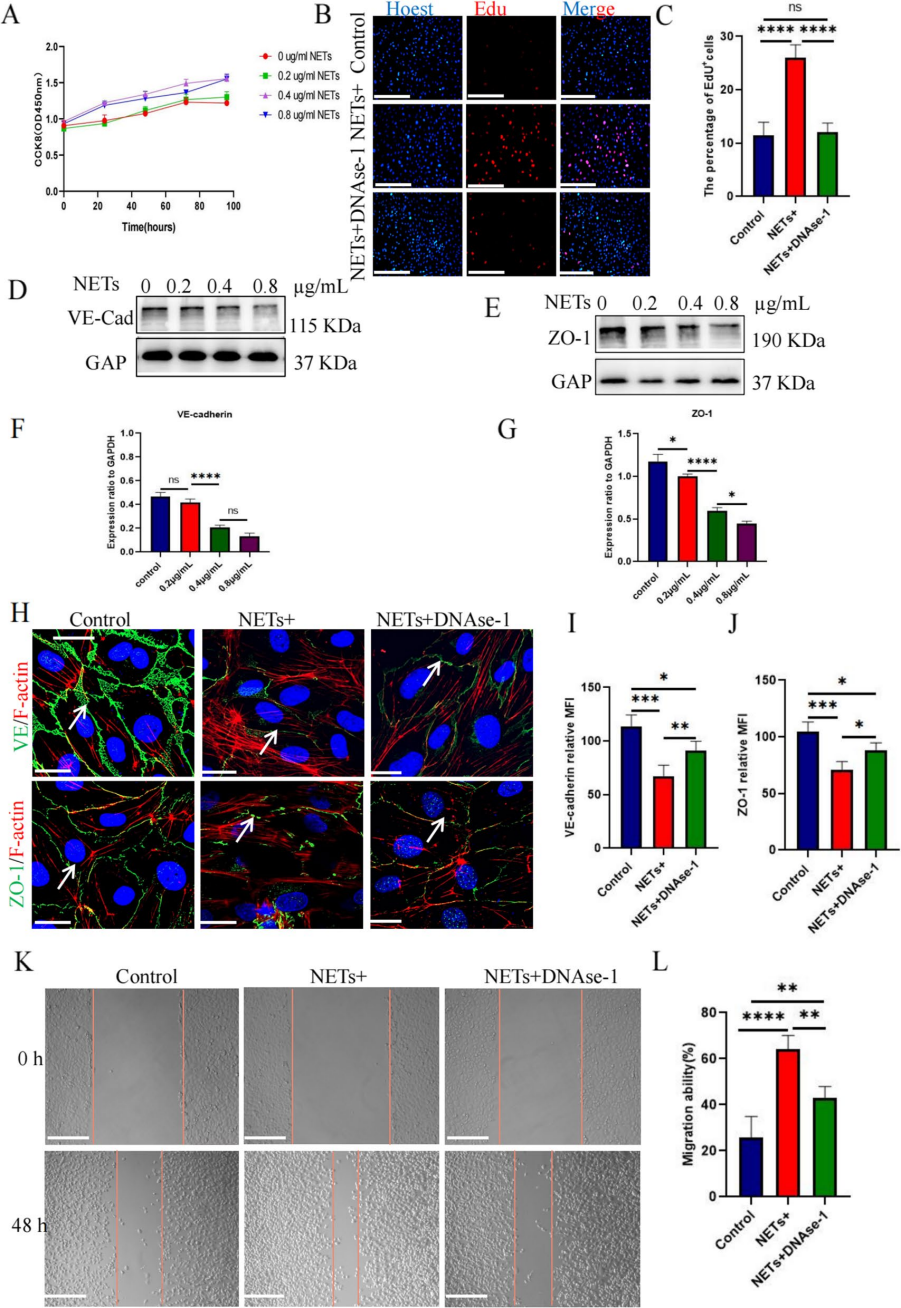
The effect of NETs on the proliferation and migration of HUVECs. **a** The proliferation ability was detected using the CCK-8 method under different NET concentrations: 0, 0.2, 0.4, and 0.8 μg/mL.** b** EdU labeling was used to detect the effect of NETs on the proliferation of HUVECs and the inhibitory effect of DNase-1. Magnification, × 20; scale bars: 50 μm; red, EdU; blue, Hoechst.** c** The HUVEC proliferation rate under EdU labeling; all values are presented as means ± SDs. ****p < 0.0001. **d**,** e** Western blotting to detect changes in VE-cadherin and ZO-1 expression under stimulation with different NET concentrations: 0, 0.2, 0.4, and 0.8 μg/mL. **f**,** g** Image J software was used to calculate the gray values of VE-cadherin and ZO-1 under different NET concentrations; *p < 0.05, ****p < 0.0001. **h** The effect of NETs on the expression of VE-cadherin and ZO-1 and the inhibitory effect of DNase-1 as observed by immunofluorescence staining. Magnification, × 20; scale bars: 50 μm; red, F-actin; green, VE-cadherin/ZO-1; blue, DAPI. **i, j** The average fluorescence intensities of VE-cadherin and ZO-1 were calculated using Image J software. All values are presented as means ± SDs. *p < 0.05; **p < 0.01; ***p < 0.001. **k** The effect of NETs on the migration of HUVECs and the inhibition of DNase-1 as evaluated using the scratch test. Magnification, × 20; scale bars: 50 μm. **l** The percentage of cell migration area was calculated using Image J software. All values are presented as means ± SDs. **p < 0.01; ****p < 0.0001. NET, neutrophil extracellular trap; DNase-1, deoxyribonuclease I; SD, standard deviation

**Fig. 7:**
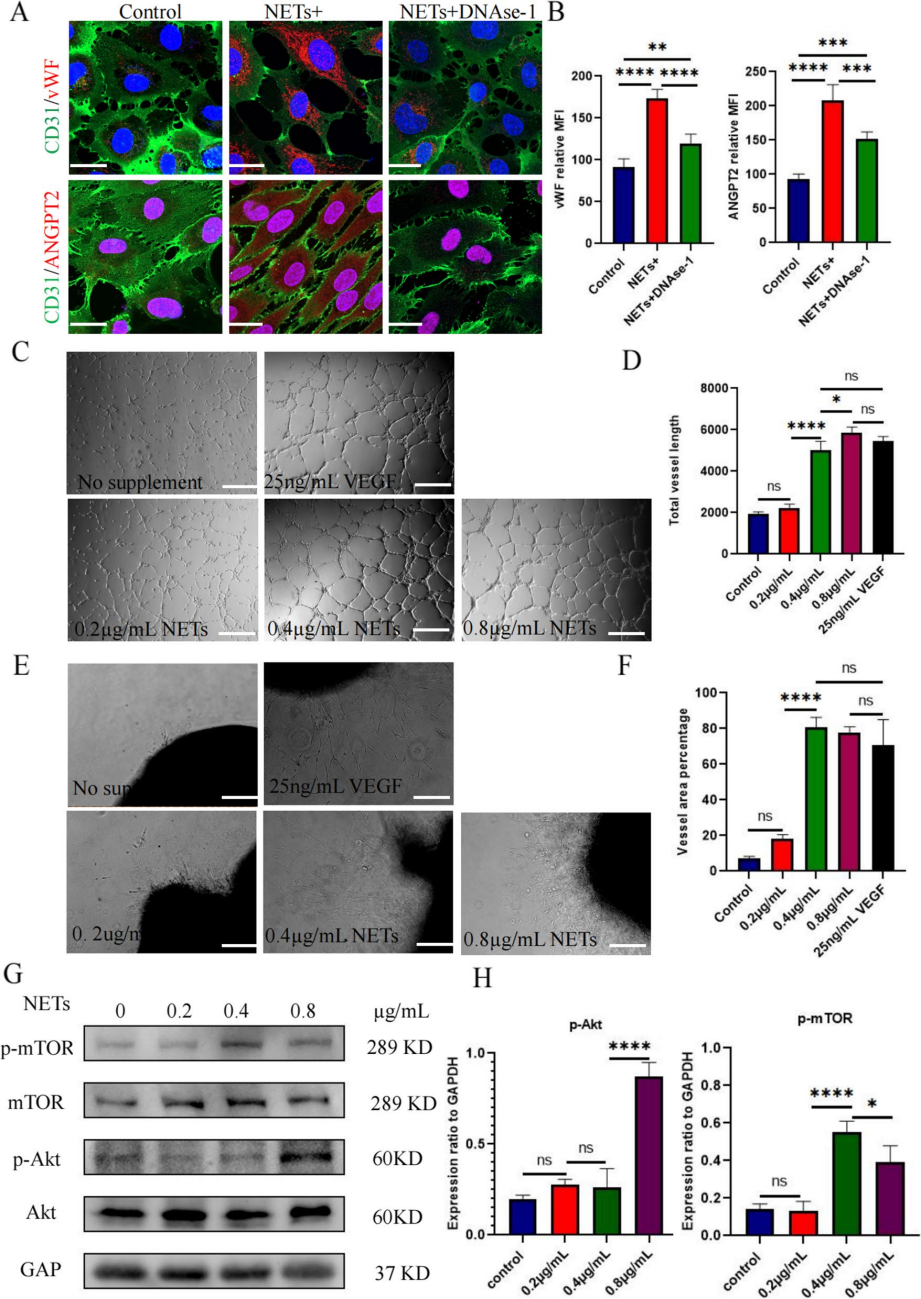
NETs activated the AKT/mTOR signaling pathway to promote angiogenesis.** a** The effect of NET-stimulated HUVECs on the injury factor vWF and pro-angiogenic factor ANGPT2 and the inhibitory effect of DNase-1 were observed via immunofluorescence. Magnification, × 20; scale bars: 50 μm; red, ANGPT2/vWF; green, CD31; blue, DAPI. **b** The average intensities of vWF and ANGPT2 were calculated using Image J software; all values are presented as means ± SDs. **p < 0.01; ***p < 0.001; ****p < 0.0001.** c** The tubule-forming ability of HUVECs with 0, 0.2, 0.4, and 0.8 μg/mL of NET stimulation and 25 ng/mL VEGF stimulation was detected via a tubule formation experiment. Magnification, × 20; scale bars: 50 μm.** d** The total length of vascular rings formed under different stimulation conditions was calculated using Image J software; all values are presented as means ± SDs. *p < 0.05; ****p < 0.0001. **e** The neovascularization ability was detected using the arterial ring germination test in nude mice with NET concentration gradients of 0, 0.2, 0.4, and 0.8 μg/mL and stimulation with 25 ng/mL VEGF. Magnification, × 20; scale bars: 50 μm. **f** Image J software was used to calculate the coverage area of vessels under different stimulation conditions; all values are presented as means ± SDs. ****p < 0.0001.** g** Western blotting was used to detect the activation and phosphorylation of the AKT/mTOR signaling pathway induced by NET-stimulated HUVECs. **h** Image J software was used to calculate the gray values of p-AKT/p-mTOR; all values are presented as means ± SDs. *p < 0.05; ****p < 0.0001. vWF, von Willebrand factor; ANGPT2, angiopoietin-2; SD, standard deviation

Upon further evaluating the effects of NETs on HUVECs, we found that endothelial cell proliferation was not obvious when the concentration of NETs was 0.2 μg/mL but increased significantly at 0.4 μg/mL, and there was no significant difference between the results at concentrations of 0.8 and 0.4 μg/mL (Fig. 6a). A concentration of 0.4 μg/mL in the EdU experiment resulted in significantly higher proliferation in the experimental group than in the control group, and this effect could be eliminated by DNase I treatment (Fig. 6b, c). After endothelial cells were stimulated with NETs in a concentration-dependent manner, western blot analysis showed that the levels of connexin VE-cadherin and the compact protein ZO-1 decreased significantly at 0.4 μg/mL NETs (Fig. 6d–g). Immunofluorescence showed that after endothelial cell stimulation with 0.4 μg/mL NETs, the intercellular space increased, and the levels of VE-cadherin and ZO-1 decreased significantly. However, DNase I treatment weakened this phenomenon (Fig. 6h-j). To determine the effects of NETs on endothelial cell migration, we carried out a scratch test. In contrast to the results in the control group, NETs significantly stimulated HUVEC migration in the experimental group (Fig. 6k, l). The tubule formation experiment showed a stronger tubule formation ability at 0.4 μg/mL NETs (Fig. 7c, d). These results suggest that NETs can directly stimulate endothelial cell proliferation, survival, and migration.

Proteomic analysis showed that NETs had no significant effect on the traditional angiogenic factor VEGF but promoted ANGPT2 production (Figs. S2a, b). Using immunofluorescence, we found that NETs stimulated ANGPT2 release from endothelial cells, and DNase I treatment weakened this phenomenon (Fig. 7a, b). We further analyzed the function of ANGPT2 through bioinformatics and found that high ANGPT2 expression was significantly associated with a poor prognosis, and its expression level was consistent with neutrophil infiltration (Fig. S2c, S2d). Patients with high ANGPT2 expression had lower tumor immune dysfunction and exclusion scores and were more likely to benefit from immunotherapy, which provides a reference to guide the use of immunosuppressants (Fig. S2e). Furthermore, we found high levels of ANGPT2 expression in the tumor tissues of gastric cancer patients and even greater levels in adjacent tissues using immunohistochemical staining; the results were statistically significant (Fig. S2f, S2g).

### NETs promote angiogenesis of arterial rings in SD female rats

To explore the process of angiogenesis in vivo, we embedded the isolated arterial rings of SD female rats into Matrigel as an in vitro model to study microvessels. We found that 0.4 and 0.8 μg/mL NETs significantly promoted vascular development, but the effect was not significantly different from that in the VEGF group; furthermore, the effect of 0.2 μg/mL NETs was not significant (Fig. 7e, f).

### NETs induce activation of the AKT/mTOR signaling pathway

To investigate the downstream signaling pathway induced by NETs in endothelial cells, we performed western blot analysis and found that coculture with NETs for 24 h increased the phosphorylation of AKT and mTOR in HUVECs. The relative increase in quantitative expression was statistically significant (Fig. 7g, h).

## Discussion

In this study, the deposition of large amounts of NETs in the tissues of gastric cancer patients was related to a worse clinical stage and abundant blood supply. We screened differentially expressed proteins induced by the effects of NETs on HUVECs in gastric cancer patients using proteomics technology and explored whether NETs promote angiogenesis in gastric cancer. TMT-MS/MS identified 6,182 differentially expressed proteins after HUVEC stimulation with NETs. Functional enrichment analysis of the identified differentially expressed proteins showed that NETs played a role in the regulation of cell metabolism, apoptosis, immune and defense responses, and other pathways in HUVECs. TMT-MS/MS results showed that endothelial cells expressed the NET-DNA receptor CCDC25, which could directly promote endothelial cell proliferation, migration, and tubule formation. The experimental model of arterial rings in vitro showed significant promotion of neovascularization. In vivo studies confirmed that NETs promoted tumor growth by increasing microvessel density, whereas DNase I inhibited NET-induced tumor angiogenesis and thus suppressed tumor progression. There is evidence that NETs play a role in angiogenesis: in pulmonary hypertensive vascular disease, NETs promote neovascularization and improve ischemia and hypoxia [34]. However, there is little evidence about the role of NETs in tumor angiogenesis.

NETs consist of chromatin from DNA fragments encapsulating granule proteins, which are released by neutrophils to capture microorganisms [35, 36]. Although the mechanism underlying the effects of NETs on cancer cells has not been fully elucidated, recent findings suggest that NETs promote cancer progression. Park et al. showed that lung metastasis may occur after 4T1 metastatic breast cancer cells are injected into the tail veins of LysM-EGFP mice, and there is extensive NET formation in lung metastatic foci. Immunofluorescence assays of lung tissue sections showed that the number of neutrophils producing NETosis significantly increased 4 days after tumor cell injection, and DNase I reduced the occurrence of lung tumor metastasis in mice [37]. For colon cancer patients who underwent hepatectomy, the 5-year survival rate of patients with elevated levels of peripheral blood NETs was significantly lower than that of patients with normal NET levels [38]. Our previous studies also confirmed that NETs can promote EMT in gastric cancer cells, and NET inhibition can reduce the occurrence of liver metastasis in animal models. An inferior vena cava embolism model in tumor-bearing mice showed a large amount of NET deposits in the thrombus. In this study, we found that greater NET deposition and the presence of abundant microvessels translated to a worse clinical stage. Clinically, neutrophils from gastric cancer patients were more likely to be activated and release NETs under the same conditions than those from healthy volunteers. Therefore, we hypothesize that NETs play an important role in the occurrence and development of gastric cancer.

Nie et al. confirmed that NETs promote the proliferation and migration of diffuse large B-cell lymphoma cells via TLR9 signaling and mediate the progression of lymphoma [39]. Although the results of the current study are consistent with those of other studies, the difference in this study lies in our finding that NETs had no significant effect on the proliferation of gastric cancer cells. Song et al. found that NET-DNA activates the intracellular ILK-β-parvin signaling pathway and enhances the motor ability of cells by binding to the transmembrane protein receptor CCDC25 on the surface of cancer cells. Knockout or blockade of the CCDC25 protein can significantly inhibit NET-DNA-mediated metastasis of cancer cells, which confirms that CCDC25 is the receptor of NET-DNA [40]. In this study, we screened the differentially expressed proteins resulting from HUVEC stimulation by NETs using the TMT-MS/MS technique and found that HUVECs also expressed CCDC25. Through immunofluorescence localization, we found that CCDC25, a transmembrane protein expressed in HUVECs, was transferred to the cytoplasm after NET stimulation, activated the AKT/mTOR axis to mediate signal transduction, and promoted HUVEC proliferation, migration, and tubule formation, thereby initiating neovascularization, improving the ischemic condition of the tumor microenvironment, and promoting tumor growth. Through further bioinformatics analysis, we found that CCDC25 was significantly associated with neutrophils in immune cell infiltration, which further verified the reliability of our results. Moreover, drug sensitivity analysis showed that gastric cancer patients with low CCDC25 expression had a better response to drugs, which is relevant information for guiding clinicians in selecting drug treatments.

Endothelial cells are some of the most silent cells in the human body, with a proliferation rate close to zero in a steady state; however, with adequate stimulation, they can re-enter the cell cycle [41]. Tight junctions play an important role in maintaining vascular integrity and controlling the permeability of the endothelial monolayer [42]. Hence, downregulated expression or loss of tight junctions can affect cell migration, proliferation, polarity, and differentiation, which may promote cancer progression. Primary breast tumors reduce the levels of tight junction-related ZO-1 proteins in endothelial cells, which are associated with metastasis in breast cancer patients [43]. MicroRNAs may also cause tight junction failure [44]. Few studies have investigated the effect of NETs on tight junctions in tumors. Wang et al. showed that NETs can damage the integrity of endothelial cells and promote permeability, thus aggravating bleeding during the treatment of acute promyelocytic leukemia. After DNase I stabilized the cytoskeletal distribution of connexin and actin, the endothelial cell permeability and erythrocyte exudation rate were significantly reduced [45]. Our previous study showed that NETs promote the release of ANGPT2 from HUVECs [46]. In the current study, we found that NETs significantly stimulated endothelial cells; decreased the expression of CD31, ZO-1, and VE-cadherin; contracted the cytoskeleton; enlarged the intercellular space; and promoted the proliferation and migration of HUVECs, whereas DNase I significantly inhibited these phenomena. Recent studies have shown that neovascularization can promote the distant metastasis of tumor cells, which is significantly related to the high permeability of HUVECs [47]. However, the main purpose of this study was to explore the role of neovascularization in promoting gastric cancer proliferation; whether neovascularization induces the distant metastasis of gastric cancer remains to be confirmed.

Tumor angiogenesis is a complex process that is regulated by tumor angiogenic factors (positive regulation) and antitumor angiogenic factors (negative regulation), and the occurrence of angiogenesis mainly depends on a balance between the two [48]. Tumor cells can release angiogenic factors, including VEGF, fibroblast growth factor, angiopoietin, epidermal growth factor, platelet-derived growth factor, and matrix metalloproteinases that promote tumor neovascularization, among which VEGF plays a decisive role in promoting endothelial cell growth and differentiation [49]. At present, targeted drugs that suppress tumor angiogenesis mainly inhibit VEGF and commonly include pazopanib and ivermus, which are used in the treatment of renal cell carcinoma [50]. Bevacizumab, which is used to treat non-small cell lung cancer, also inhibits VEGF [51]. In addition, panizumab, cetuximab, and trastuzumab are all targeted inhibitors of VEGF. Therapeutic effects have been demonstrated in the clinical application of VEGF inhibitors; however, the occurrence of drug resistance greatly reduces the effectiveness of these drugs [52]. Therefore, other targets that promote angiogenesis should be inhibited via drug combinations to better antagonize tumor angiogenesis. In this study, we found that the effect of NETs on VEGF induction in HUVECs was not significant. In vitro murine arterial ring neovascularization and lumen formation experiments showed that NETs significantly promoted neovascularization but had no synergistic effect with VEGF. The subcutaneous tumor model in nude mice revealed lower tumor volume, tumor growth rate, and microvessel density among mice treated with DNase I than in the control group. Therefore, we believe that targeting NETs may be an effective strategy for antagonizing tumor neovascularization and inhibiting tumor growth.

This study has some limitations. First, we utilized single-center samples, and more samples are needed to verify our findings in future studies and to obtain results that are more widely representative. Second, we investigated gastric cancer, and the role of NETs in other malignant tumors of the digestive tract needs to be further explored. In addition, the effect of CCDC25 expression on chemotherapeutic drugs in gastric cancer is based on the Shengxin database, which needs to be verified and analyzed via more complete basic trials and clinical data.

## Conclusion

In this study, we found and verified the expression of the NET-DNA receptor CCDC25 in ECs. NETs produced in the gastric cancer microenvironment act on ECs to achieve neovascularization and promote the progression of gastric cancer. Therefore, targeting NETs may be a potential therapeutic strategy for antiangiogenesis and can improve the current predicament of disease progression caused by drug resistance.

## Supplementary Information

**Additional file 1: Table S1.** Differential proteins identified by TMT-MS/MS.

**Additional file 2: Figure S1. **Effect of NETs on the proliferation of the gastric cancer cell lines MKN-1 and HGC27.The proliferation of the gastric cancer cell lines MKN-1 and HGC27 was detected using CCK-8 and the plate cloning experiment. NET, neutrophil extracellular trap.

**Additional file 3: Figure S2.** Bioinformatics analysis of the biological function of ANGPT2 in gastric cancer.TMT-MS/MS and PRM techniques were used to verify that NETs promote the release of ANGPT2 from HUVECs.Immunocyte infiltration analysis showed that the expression of ANGPT2 was positively correlated with neutrophil infiltration.The relationship between ANGPT2 expression and the survival time was analyzed using the OS curve.The relationship between the Tumor Immune Dysfunction and Exclusion score and ANGPT2 expression level.Immunohistochemical staining was used to analyze the expression of ANGPT2 in tumor tissues and corresponding paracancerous tissues.The area covered by ANGPT2 expression in immunohistochemical staining. Magnification, ×20; scale bars: 50 μm. TMT-MS/MS, tandem mass tag-tandem mass spectrometry, NET, neutrophil extracellular trap; HUVECs, human umbilical vein endothelial cells

**Additional file 4.**

## Data Availability

The datasets used and/or analyzed during the current study are available from the corresponding author upon reasonable request.

## Abbreviations

ANGPT2: Angiopoietin-2
CCK-8: Cell counting kit-8
DAPI: 4′,6-Diamidino-2-phenylindole
EMT: Epithelial-mesenchymal transformation
HMVEC: Human pulmonary microvascular endothelial cell
HRP: Horseradish peroxidase
HUVEC: Human umbilical vein endothelial cell
MPO: Myeloperoxidase
NET: Neutrophil extracellular trap
PBS: Phosphate-buffered saline
VEGF: Vascular endothelial growth factor
vWF: Von Willebrand factor
