# Supplementary figures and images for: Neutrophil extracellular traps promote angiogenesis in gastric cancer
Source: Cell Commun Signal. 2023 Jul 21;21:176. doi: 10.1186/s12964-023-01196-z (PMC10362668; doi:10.1186/s12964-023-01196-z)

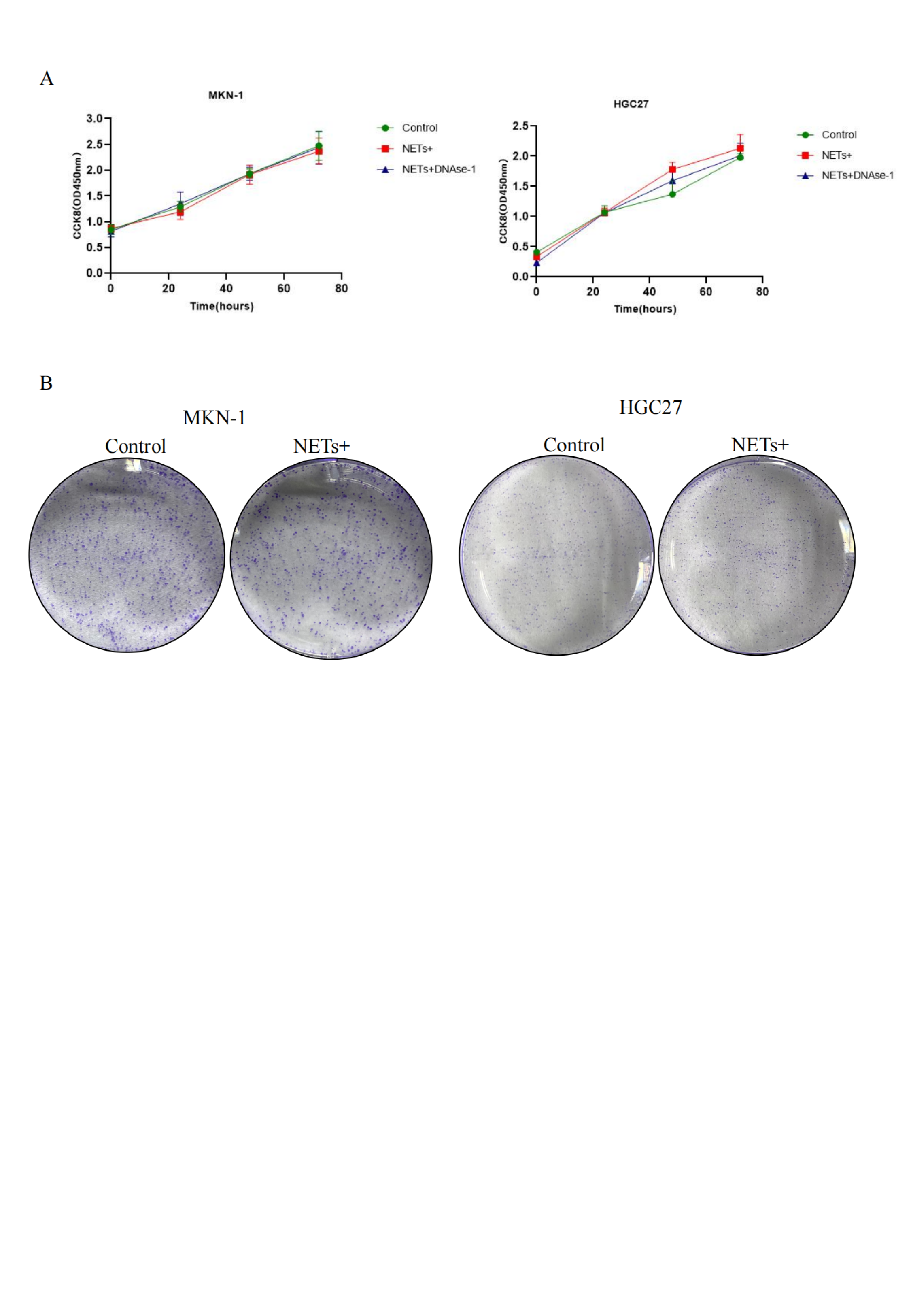

Supplement: Supplementary file 3 — Additional file 2: Figure S1. Effect of NETs on the proliferation of the gastric cancer cell lines MKN-1 and HGC27.The proliferation of the gastric cancer cell lines MKN-1 and HGC27 was detected using CCK-8 and the plate cloning experiment. NET, neutrophil extracellular trap. [file 12964_2023_1196_MOESM2_ESM.tif]

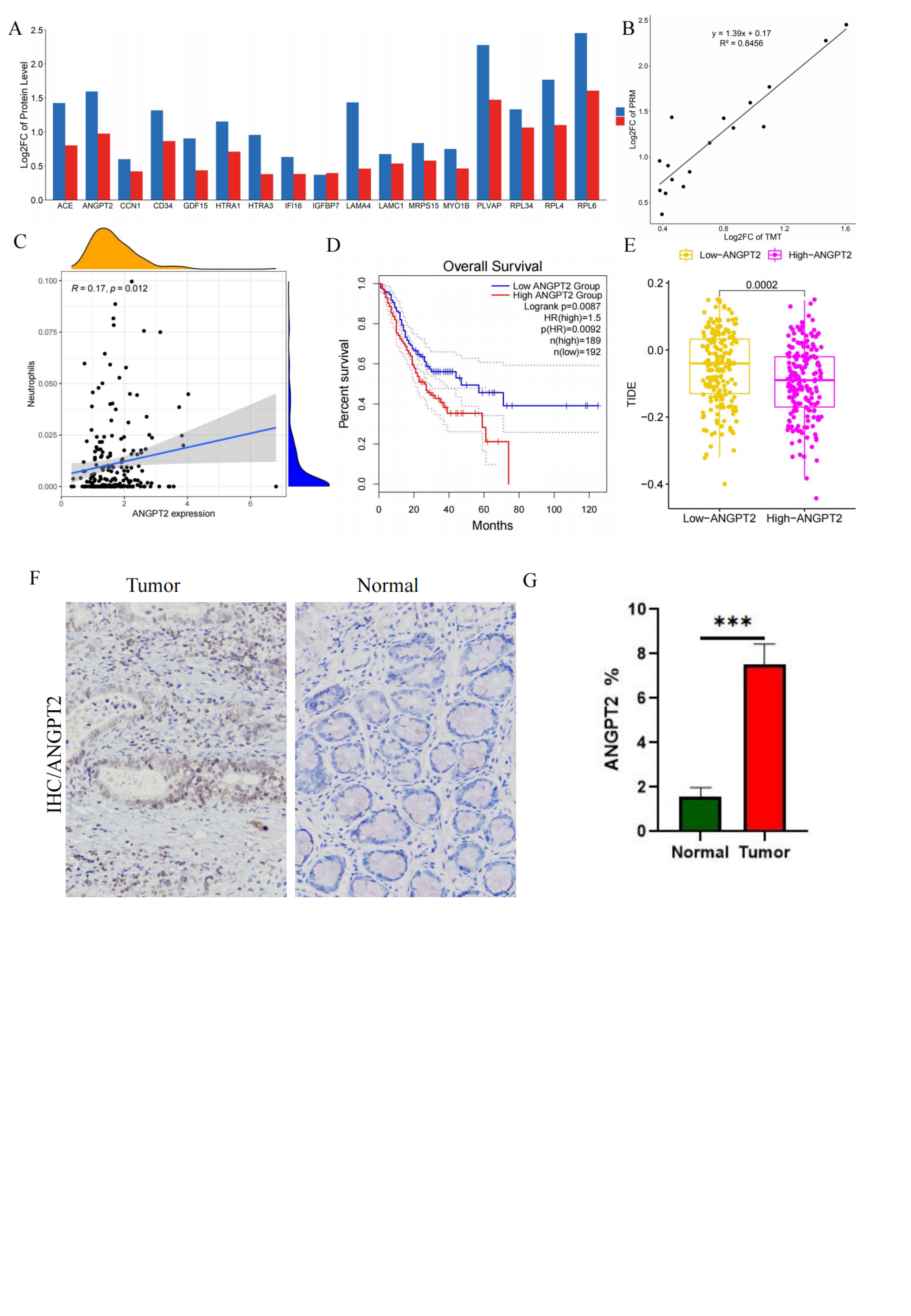

Supplement: Supplementary file 4 — Additional file 3: Figure S2. Bioinformatics analysis of the biological function of ANGPT2 in gastric cancer.TMT-MS/MS and PRM techniques were used to verify that NETs promote the release of ANGPT2 from HUVECs.Immunocyte infiltration analysis showed that the expression of ANGPT2 was positively correlated with neutrophil infiltration.The relationship between ANGPT2 expression and the survival time was analyzed using the OS curve.The relationship between the Tumor Immune Dysfunction and Exclusion score and ANGPT2 expression level.Immunohistochemical staining was used to analyze the expression of ANGPT2 in tumor tissues and corresponding paracancerous tissues.The area covered by ANGPT2 expression in immunohistochemical staining. Magnification, ×20; scale bars: 50 μm. TMT-MS/MS, tandem mass tag-tandem mass spectrometry, NET, neutrophil extracellular trap; HUVECs, human umbilical vein endothelial cells [file 12964_2023_1196_MOESM3_ESM.tif]
